# Interpretability and Minimal Important Change of the Greek Quality of Life in Hand Eczema Questionnaire (QOLHEQ)

**DOI:** 10.1111/cod.70006

**Published:** 2025-08-12

**Authors:** Robert F. Ofenloch, Christian Apfelbacher, Dimitra Koumaki

**Affiliations:** ^1^ Division of Occupational Dermatology, Department of Dermatology University Hospital Heidelberg Heidelberg Germany; ^2^ Department of General Practice and Health Service Research University Hospital Heidelberg Heidelberg Germany; ^3^ Institute of Social Medicine and Health Systems Research Otto von Guericke University Magdeburg Magdeburg Germany; ^4^ Dermatology Department University Hospital of Heraklion Heraklion Crete Greece

**Keywords:** hand eczema, health‐related quality of life (HRQOL), interpretability, validation

The Quality of Life in Hand Eczema Questionnaire (QOLHEQ) is a hand eczema (HE) specific instrument used to assess health‐related Quality of Life (HRQOL) in four subdomains [1]. The QOLHEQ is available in 10 language versions; one international and five national validation studies have already been published [1, 2, 3, 4, 5, 6]. Score banding and minimal important change (MIC) have not been established yet for the Greek version of the QOLHEQ.

## 1 Methods

We investigated the interpretability and minimal important change (MIC) of the Greek version of the QOLHEQ. The QOLHEQ was used together with a global anchor question as well as four anchors specific to the four QOLHEQ subdomains (according to [7]) in a longitudinal manner at (i) baseline, (ii) after 1–3 days (stable HE), and (iii) after 4–6 weeks (HE changed). Change in HE severity was assessed using a patient‐reported global rating of change scale. Cohen's weighted kappa was used to identify optimal score bands for the total QOLHEQ score and its subscales.

## 2 Results

Data were collected from 300 patients aged 18 to 89 years (mean 45.6 ± 14.2; 65.3% female). In all analyses, the amount of missing values was < 2%. According to PGA, 37.3% of patients had moderate and 21.3% had severe HE. Severity was nearly normally distributed in the sample. All anchor questions exhibited the strongest correlation with their respective QOLHEQ domains, ranging from *r* = 0.50–0.82. The optimal score banding for the total score was found to be:

- No impairment: < 15
- Slight impairment: 15–25
- Moderate impairment: 26–44
- Strong impairment: 45–73
- Very strong impairment: > 73

Score bands for the individual subscales were identified as follows (no impairment, slight, moderate, strong and very strong):

Symptoms: 0–2, 3–7, 8–13, 14–20 and ≥ 21.

Emotions: 0–3, 4–10, 10–15, 16–20 and ≥ 21.

Functioning: 0–3, 4–12, 13–15, 16–21 and ≥ 22.

Treatment and prevention: 0–3, 4–7, 8–15, 16–22 and ≥ 23. The minimal important change (MIC) was determined through anchor‐ and distribution‐based methods, with a total score MIC identified as 7.0 points while the smallest detectable change was 5.6 points. Subscale‐specific MIC (SDC) values were as follows: symptoms 1.7 (1.6), emotions 2.3 (1.9), functioning 3.1 (2.3) as well as treatment and prevention 1.9 (1.6).

## 3 Discussion

Our findings provide practical thresholds for interpreting the Greek QOLHEQ scores, enabling clinicians and researchers to more clearly assess the severity of HRQOL impairment. The established MIC facilitates the evaluation of meaningful clinical changes in patients' conditions, thus improving patient management and enhancing comparability with international studies. Our results indicate that scores from the Greek QOLHEQ version can be interpreted very similarly to the Dutch [3] and German [1] language versions, where banding studies have already been performed. Compared to other versions, the Greek MIC is much lower. The study was conducted at the University Hospital of Heraklion, Crete, with mostly fishermen, agricultural workers and nurses in our sample. The lower MIC may reflect a more homogeneous patient group with similar aetiology and prognosis.

## Conflicts of Interest

R.F.O. and D.K. declare no conflicts of interest. C.A. received institutional funding from Bionorica SE and Dr. Wolff Group and consultancy fees from EFCNI, Bionorica SE, Dr. Wolff Group, Rheacell, Sanofi, Pfizer, LEOPharma and Incyte.

## Data Availability

The data that support the findings of this study are available on request from the corresponding author. The data are not publicly available due to privacy or ethical restrictions.
